# An Overview of the Mechanisms Against “*Candidatus* Liberibacter asiaticus”: Virulence Targets, Citrus Defenses, and Microbiome

**DOI:** 10.3389/fmicb.2022.850588

**Published:** 2022-03-10

**Authors:** Chuanyu Yang, Veronica Ancona

**Affiliations:** Department of Agriculture, Agribusiness, and Environmental Sciences, Citrus Center, Texas A&M University-Kingsville, Weslaco, TX, United States

**Keywords:** HLB, unculturable bacteria, *C*Las, citrus defenses, HLB-tolerance

## Abstract

Citrus Huanglongbing (HLB) or citrus greening, is the most destructive disease for citrus worldwide. It is caused by the psyllid-transmitted, phloem-limited bacteria “*Candidatus* Liberibacter asiaticus” (*C*Las). To date, there are still no effective practical strategies for curing citrus HLB. Understanding the mechanisms against *C*Las can contribute to the development of effective approaches for combatting HLB. However, the unculturable nature of *C*Las has hindered elucidating mechanisms against *C*Las. In this review, we summarize the main aspects that contribute to the understanding about the mechanisms against *C*Las, including (1) *C*Las virulence targets, focusing on inhibition of virulence genes; (2) activation of citrus host defense genes and metabolites of HLB-tolerant citrus triggered by *C*Las, and by agents; and (3) we also review the role of citrus microbiome in combatting *C*Las. Finally, we discuss novel strategies to continue studying mechanisms against *C*Las and the relationship of above aspects.

## Introduction

Citrus Huanglongbing (HLB), or citrus greening, is the most destructive citrus disease worldwide. It is associated with three species of fastidious, phloem-restricted α-proteobacteria: “*Candidatus* Liberibacter asiaticus” (*C*Las), “*Candidatus* Liberibacter americanus”(*C*Lam), and “*Candidatus* Liberibacter africanus” (*C*Laf), which are transmitted by the psyllids *Diaphorina citri* or *Trioza erytreae* (Jagoueix et al., 1994; Bové, 2006; Gottwald, 2010). *C*Las is the most prevalent species found in commercial citrus production regions, including the United States, China, and Brazil (Jagoueix et al., 1994; Bové, 2006; Gottwald, 2010; Bassanezi et al., 2020; Zhou, 2020). HLB symptomology include yellowing of shoots, blotchy mottled leaves, corky veins, malformed and discolored fruits, premature fruit drop, root loss, and eventually tree death (Wang and Trivedi, 2013; Blaustein et al., 2018). Unfortunately, no commercial citrus varieties are resistant to HLB.

The HLB epidemic has affected all major citrus growing regions in the world (Hodges and Spreen, 2012; Kumagai et al., 2013; da Graça et al., 2015; Graham et al., 2020). In the United States, Florida has been the most affected citrus producing state. Since HLB arrival in 2005 (Bové, 2006), citrus production in Florida has decreased by 74% (Singerman and Rogers, 2020). Production losses due to HLB have resulted in the reduction of citrus growers from 7,389 in 2002 to 2,775 in 2017, juice processing facilities from 41 in 2003/2004 to 14 in 2016/2017, and packinghouses from 79 to 26 during the same period (Singerman and Rogers, 2020). In China, HLB was first reported in Guangdong province nearly a century ago (Reinking, 1919). To date, HLB has occurred in 10 provinces in China, including Guangdong, Guangxi, Fujian, Zhejiang, Jiangxi, Hunan, Guizhou, Hainan, and Sichuan. Especially, citrus production in Guangdong, Guangxi, and Fujian have been affected by HLB for a long time (Zhou, 2020). In Brazil, HLB was first reported in São Paulo State in 2004 (Coletta-Filho et al., 2004). After the first HLB outbreak, the disease spread to the States of Minas Gerais, Paraná and Mato Grosso do Sul, causing reduction of citrus production (Bassanezi et al., 2020).

Currently, many strategies have been developed for HLB mitigation, including application of antimicrobials (Zhang et al., 2011a, 2012, 2014, 2021; Hu and Wang, 2016; Hu et al., 2018; Yang et al., 2018), thermotherapy (Hoffman et al., 2013; Fan et al., 2016; Yang et al., 2016b; Doud et al., 2017; Ghatrehsamani et al., 2019; Vincent et al., 2019), macro-and micronutrients (Spann and Schumann, 2009; Gottwald et al., 2012; Rouse et al., 2017; Mattos-Jr et al., 2020; Dong et al., 2021; Zhou et al., 2021), plant defense inducers (Canales et al., 2016; Li et al., 2016, 2019, 2021a; Hu et al., 2018; Wang, 2021), control of the insect vector (Grafton-Cardwell et al., 2013; Boina and Bloomquist, 2015; Cocuzza et al., 2017; Pierre et al., 2021), biocontrol (Trivedi et al., 2011; Hopkins and Wall, 2021; Nan et al., 2021; Poveda et al., 2021), and eradication of HLB symptomatic citrus trees (Bassanezi et al., 2013; Yuan et al., 2020). However, these strategies have shown limited success in field applications and effective HLB management remains a challenge. Three-pronged approach including control of the psyllid vector, aggressive removal of infected trees to reduce sources of the disease, and planting with HLB-free nursery stock, has proven successful in China and Brazil, and has resulted in drastic reductions in the proportion of symptomatic trees (Bové, 2006). While this approach was advocated early on in Florida’s HLB outbreak, it was deemed to be too expensive by most producers, who instead decided to maintain symptomatic trees as long as they were bearing usable fruit (Hall and Gottwald, 2011). In addition, non-uniform distribution of *C*Las within citrus tree (Tatineni et al., 2008; Li et al., 2009) makes early detection of *C*Las very difficult, which is crucial for the management of citrus HLB. Thus, breeding for HLB disease-resistance may provide the most effective and sustainable solution to combat HLB (Bové, 2006).

In order to develop novel and effective strategies to suppress HLB, it is important to understand the virulence mechanisms employed by *C*Las to be able to elucidate potential targets against the pathogen. In this review, we describe the different virulence mechanisms of *C*Las and strategies used to identify virulence inhibitors. We also discuss the role of plant defenses in conferring HLB tolerance and the potential role of the citrus microbiome against *C*Las. We conclude with a discussion about the new pathways for studying this uncultured bacterial pathogen.

## *C*Las Virulence Targets

Most insights of *C*Las virulence and biological processes are derived from the genome sequence of *C*Las (Duan et al., 2009), and other related Liberibacters (Coyle et al., 2018). Many putative virulence factors have been identified by utilizing surrogate models, and several strategies also have been developed for targeting these virulence genes associated with *C*Las pathogenicity and survival.

## Secretion Systems and Effectors

Systems capable of secreting bacterial proteins, called effectors, into host cells are among the most important virulence factors of bacterial pathogens. Protein effectors often suppress plant defenses or manipulate developmental processes within the host to benefit the pathogen (Jones and Dangl, 2006). *C*Las encodes type I secretion systems (T1SS), a complete general secretory pathway (Sec), and an autotransporter type V secretion system (T5SS), but lacks other secretion systems (Duan et al., 2009; Fagen et al., 2014; Wulff et al., 2014; Wang et al., 2017). The Sec machinery facilitates the majority of proteins transport across the cytoplasmic membrane and is essential for bacterial viability (Segers and Anné, 2011). The Sec apparatus also secretes important virulence factors in some plant-pathogenic bacteria. It has been reported that *C*Las has at least 86 proteins with functional Sec-dependent secretion signals (Prasad et al., 2016). Many of these proteins, also called Sec-delivered effectors (SDEs) are highly conserved in *C*Las genomes and exhibit differential expression patterns in the citrus host and the psyllid vector (Thapa et al., 2020). *C*Las Sec-delivered effector 1 (SDE1, CLIBASIA_05315), is conserved across *C*Las isolates with a typical Sec-dependent secretion signal (Pitino et al., 2016; Prasad et al., 2016; Pagliaccia et al., 2017). SDE1 is highly expressed in citrus relative to psyllid, indicating a plausible role in *C*Las colonization of citrus and HLB disease progression (Yan et al., 2013). SDE1 inhibits the enzymatic activity of citrus papain-like cysteine proteases (PLCPs), which regulate multiple processes in plants, including defense against microbial pathogens (Clark et al., 2018). Other studies also suggested that SDE1 contributes to *C*Las colonization and the development of leaf yellowing symptoms, possibly by promoting premature senescence in citrus (Pitino et al., 2016; Clark et al., 2020). Although, there is no evidence that targeting effectors would lead to *C*Las suppression, targeting the Sec system could inhibit protein translocation and have a significant effect on *C*Las virulence and survival. The SecA ATPase drives protein translocation when it is bound to the SecYEG complex (Economou and Wickner, 1994; Van den Berg et al., 2004). Based on characteristics of SecA, 20 small molecules against *C*Las were identified by molecular docking *in silico*, and five of these compounds were confirmed to have antimicrobial activity *in vitro* using *Agrobacterium tumefaciens* as culturable model (Akula et al., 2012). Using a similarity search methodology, 11 compounds were identified based on the five SecA inhibitors (Hu et al., 2016). Although these 11 compounds had poor aqueous solubility, they were coupled in a micro-emulsion to assess their antimicrobial activities on eight bacteria phylogenetically related to *C*Las (*A. tumefaciens*, *Liberibacter crescens*, *Rhizobium etli*, *Bradyrhizobium japonicum*, *Mesorhizobium loti*, and *Sinorhizobium meliloti*). The inhibitions obtained from these compounds were similar to those described for streptomycin (Hu et al., 2016). Thus, the compounds targeting SecA, could also inhibit protein translocation in *C*Las and have a significant effect on HLB suppression.

## Transcriptional Regulators

The reduced genome of *C*Las has a small number of transcriptional regulators that if targeted by high affinity inhibitors could result in strong reduction of *C*Las fitness and survival (Table 1). For instance, the transcriptional regulator *PrbP* was identified and the genome of *C*Las and was shown to bind to specific promoter regions of *C*Las DNA as well as to interact with *RpoB*, the β subunit of RNA polymerase (Gardner et al., 2016). *In vitro* screening of chemical compounds that target this gene identified one compound, tolfenamic acid, that inhibited PrbP/RpoB interaction and *PrbP* DNA binding. Further evaluation showed that tolfenamic acid inhibited *in vitro* growth of *L. crescens*, affected viability of *C*Las in citrus leaf-soaking assays, and reduced *C*Las titers in infected seedlings causing the recovery of roots and canopy tissues (Gardner et al., 2016). The antimicrobial activity of Tolfenamic acid against *C*Las might be the result of targeting key regulatory components that inhibit multiple pathways for bacterial survival.

**Table 1:** Transcriptional regulators in uncultured bacteria *Candidatus* Liberibacter and inhibitors found in surrogate models for screening chemicals targeted the gene.

Transcriptional regulators	Function	Surrogate bacterial models	Inhibitors	References
*LdtR*	Controlling the expression of nearly 180 genes, distributed in processes such as cell motility, cell wall biogenesis, energy production, and transcription.	*Sinorhizobium meliloti*, and *Liberibacter crescens*	Benzbromarone, phloretin, hexestrol etc.	Pagliai et al., 2014; Barnett et al., 2019
*PrbP*	Regulating gene expression through interactions with the RNA polymerase β-subunit and a specific sequence on the promoter region	*Liberibacter crescens* and *Escherichia coli*	Tolfenamic acid	Gardner et al., 2016
*VisNR*	Regulate the expression of the pilin gene *flp3* involved in adhesion and psyllid colonization	*Sinorhizobium meliloti*	Bortezomib, Chemdiv C549-0604, and Chemdiv D244-0326 etc.	Sourjik et al., 2000; Andrade and Wang, 2019; Barnett et al., 2019
*RpoH*	Alternative sigma factor mediating stress responses including heat, acid, hydrogen peroxide, stationary phase growth, and envelope disrupting agents	*Sinorhizobium meliloti*	Rosiglitazone	Mitsui et al., 2004; de Lucena et al., 2010; Barnett et al., 2012, 2019

*LdtR* belongs to the MarR family transcription regulator and it has been linked to the regulation of more than 180 genes in *Liberibacter* species (Pagliai et al., 2017). In *S. meliloti*, mutation of *LdtR* resulted in morphological changes and reduced tolerance to osmotic stress. Small molecules including benzbromarone that targeted at *LdtR* were identified that caused a phenotype in *S. meliloti* and *L. crescens* similar with the insertional mutants (Pagliai et al., 2014). These small molecules were then assessed *via* a citrus shoot assay and shown to decrease the expression of *LdtR* and a gene regulated by *LdtR* potentially involved in cell wall biosynthesis. Therefore, application of small molecules that target *LdtR*, as a potential treatment option against citrus HLB.

As inhibition of transcriptional regulators provide an alternative method for mitigating *C*Las and HLB, a synthetic, high-throughput screening system to identify molecules that target *C*Las transcriptional regulators was developed (Barnett et al., 2019). This system used the closely related model bacterium, *S. meliloti*, as a heterologous host for expression of the *C*Las transcriptional activator, the activity of which was detected through expression of an enhanced green fluorescent protein (EGFP) gene fused to a target promoter. Around 120,000 compounds were screened by this system to target regulators including *LdtR*, *RpoH*, and *VisNR* and compounds that inhibited regulator activity were selected as candidate compound for combating HLB (Barnett et al., 2019). *C*Las sigma factor *RpoH* is most similar to *RpoH1* in *S. meliloti* (72% identity), which mediate response to various stressors, including heat, acid, hydrogen peroxide, stationary phase growth, and envelope disrupting agents (Mitsui et al., 2004; de Lucena et al., 2010; Barnett et al., 2012). *VisN* and *VisR*, members of the LuxR transcriptional factor family, negatively regulate the expression of the *C*Las pilin gene *flp3*, which is associated with bacterial adherence and psyllid colonization (Andrade and Wang, 2019). Thus, targeting transcriptional regulators is a potential strategy for reducing *C*Las fitness and HLB mitigation.

## Role of Prophage in *C*Las Survival

A prophage, also considered as a temperate phage, can integrate into the circular bacterial DNA chromosome, continuing this lysogenic cycle for as long as host physiology remains stable. However, stresses such as heat, UV light, starvation, or chemicals like antibiotics, which cause DNA damage to bacterial cells, activate the “SOS” stress response inducing the excision of phage DNA from the host (Oppenheim et al., 2005). Three prophage regions have been identified in *C*Las and have been classified as SC1, SC2, and SC3, based on genomic data (Zhang et al., 2011b; Zheng et al., 2016, 2018). SC1 carries putative lytic cycle genes, as phage particles in the phloem of infected periwinkle have been observed by transmission electron microscopy, although phage particles have not been observed in citrus (Fleites et al., 2014). SC2 lacks lytic cycle genes and can be integrated in the *C*Las genome or replicate as an excision plasmid prophage (Zhang et al., 2011b). Study of Zhang (2011b) indicated that SC1 and SC2 also encode multiple virulence factors that might contribute to the pathogenicity of *C*Las. Two predicated peroxidases are encoded by SC1 and SC2, which might detoxify *C*Las against reactive oxygen species (ROS), including superoxide radicals, hydrogen peroxide, and hydroxyl radicals. SC1 and SC2 also encode two predicated adhesins, which might be useful in transmission by psyllid (Zhang et al., 2011b). SC3 is not capable of reproduction *via* the lytic cycle. A restriction-modification (R-M) system of SC3 was speculated to play a role against Type 1 prophage-phage invasion (Zheng et al., 2018). The involvement of SC3 in survive of *C*Las still needs to be investigated.

Study of Ding et al. (2018) demonstrated that the relative copy number of both prophage SC1 and SC2 increased in HLB-affected host plants (citrus and periwinkle), in response to heat and antibiotic (tetracycline) treatments. These results suggest a potential mechanism for the activity of heat treatment and antibiotics against HLB through induction of *C*Las prophages causes lysis of *C*Las bacteria, reducing *C*Las population and mitigating HLB symptoms in citrus trees (Ding et al., 2018). Therefore, understanding the factors that trigger the lytic cycle in *C*Las prophages can provide a potential control strategy of citrus HLB.

## Mechanisms of HLB-Tolerant Citrus to *C*Las

**Figure 1:**
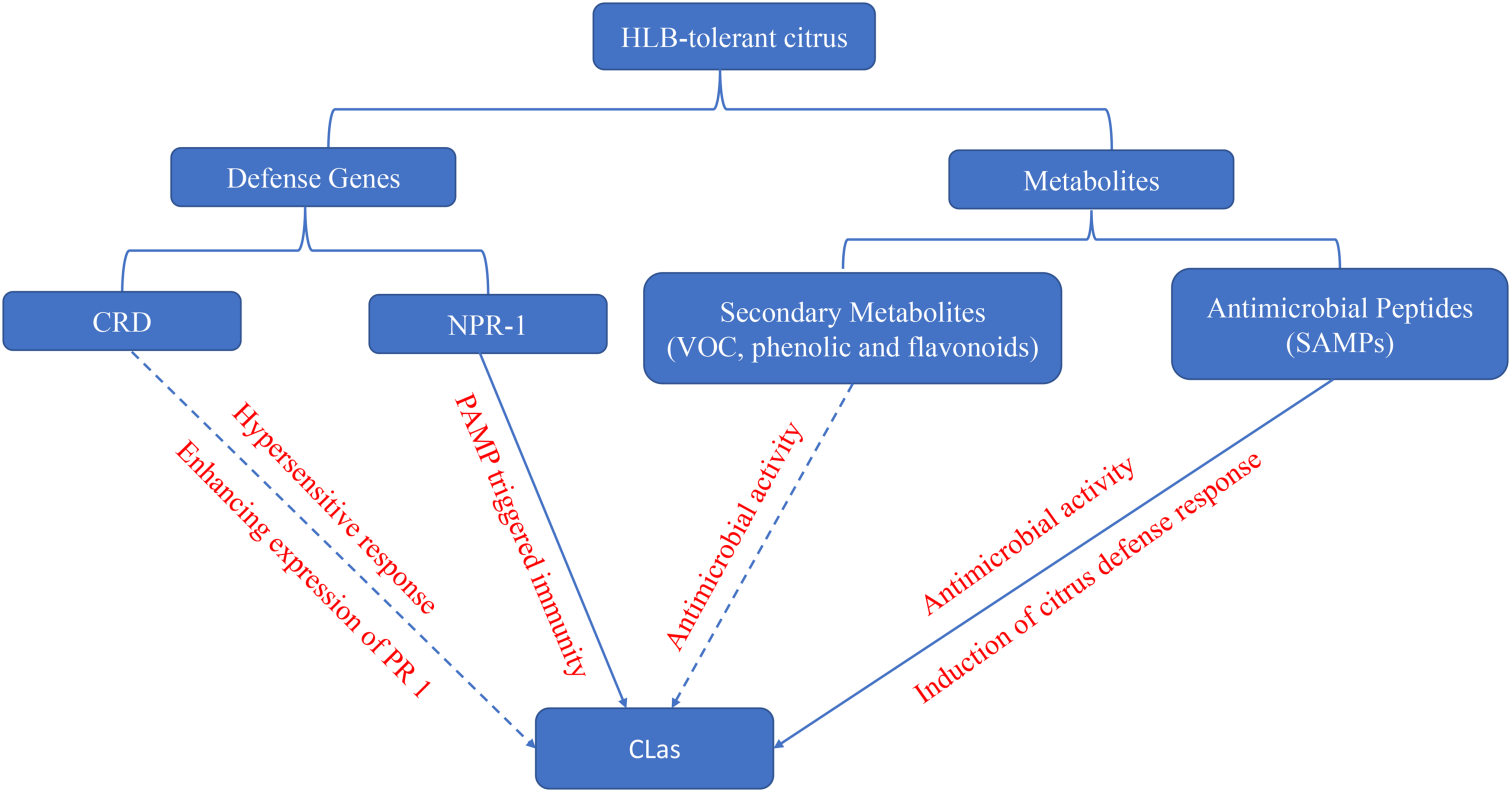
The potential mechanism of defense genes and metabolites in HLB-tolerant citrus against *C*Las. Solid line indicates that the functions were confirmed in citrus, and dash line indicates that the function were just confirmed in other species.

Citrus Huanglongbing affects all commercial citrus varieties, citrus species, and relatives (Bové, 2006). Nevertheless, several citrus cultivars and relatives have shown tolerance to *C*Las, and many studies have deciphered the mechanism of these tolerance to HLB (Table 2). Here, we would discuss host defense genes and metabolites against *C*Las (Figure 1).

**Table 2:** The mechanisms of Citrus Huanglongbing (HLB)-tolerant citrus elucidated by multi-omics approaches.

Citrus genotypes	Putative tolerance mechanisms of citrus to HLB	References
*Poncirus trifoliata* and *hybrids*	*Constitutive disease resistance 1* (*CDR1*) genes activate *PR1* expression Downregulation of gibberellin (GA) synthesis and the induction of cell wall strengthening *Poncirus trifoliata* hybrids (US-942) have a stronger defense response, more efficient nutrient uptake and increased accumulation of secondary metabolites, flavonoids, phenolics, and volatile organic compounds (VOC). Increased accumulation of phenylalanine, tyrosine, and tryptophan, and some sugars such as mannose, and α-D-mannopyranoside which are important in secondary metabolite biosynthesis and reduction of availability of essential sugars for *Candidatus* Liberibacter asiaticus (*C*Las) survival	Folimonova et al., 2009; Albrecht and Bowman, 2011, 2012; Killiny and Hijaz, 2016; Killiny, 2017; Curtolo et al., 2020; Huang et al., 2021b
Ichang papeda (*Citrus ichangensis* “2586”)	Carbohydrate metabolism, photosynthesis process, and amino acids are not activated during *C*Las infection, which may suppress HLB development Upregulation of genes involved in secondary metabolism, such as the isoprenoid and flavonoid biosynthesis pathways	Wu et al., 2020
“Jackson” grapefruit (*Citrus paradisi* Macf)	Increased expression of *NPR1*-like genes and secondary metabolite pathways	Wang et al., 2016
Mexican lime (*Citrus aurantifolia*)	Increase expression of genes related to cell wall, secondary metabolism, transcription factors, signaling, and redox reactions	Arce-Leal et al., 2020
Rough lemon (*Citrus jambhiri*)	Upregulation of genes involved in maintaining or recovering of phloem transport activity and possible enhancement of stress tolerance	Fan et al., 2012
Kaffir lime (*Citrus hystrix*)	Upregulation of genes involved in cell wall metabolism and secondary metabolism Increased expression of peroxidases, Cu/Zn-SOD, and *POD4* genes	Zou et al., 2019
Sydney hybrid (*Microcitrus virgata*)	Strong defense response upon *C*Las infection, more efficient nutrient uptake and increased accumulation of secondary metabolites, flavonoids, phenolics, and VOC	Huang et al., 2021a,b
Australian finger lime (*Microcitrus australiasica*)	Production of stable antimicrobial peptides, induction of defense responses such as salicylic acid (SA) biosynthesis, phenylpropanoid pathways, and defense genes	Huang et al., 2021a
Volkamer lemon (*Citrus Volkameriana*)	Upregulation of four glutathione-S-transferases proteins involved in radical ion detoxification	Martinelli et al., 2016
Lisbon lemon (*Citrus limon*)	Upregulation of genes involved in defense responses	Ramsey et al., 2020
Curry leaf [*Murraya koenigii* (L.) Spreng]	High level of phenolics and flavonoids with antimicrobial activity	Killiny et al., 2017; Hijaz et al., 2020
LB8-9 Sugar Belle {“Clementine” mandarin (*Citrus reticulata*) × “Minneola” tangelo [(*Citrus x Tangelo*), “Duncan” grapefruit (*Citrus paradisi*) × “Dancy” tangerine (*C. reticulata*)]}	Increased accumulation of phenolics, flavonoids, and VOCs with known antimicrobial activity such as aldehydes, monoterpenes, and sesquiterpenes Increase accumulation of plant hormones responsible for plant growth and phloem regeneration	Killiny et al., 2017; Deng et al., 2021; Suh et al., 2021

## Citrus Defense Genes Involved in Combating *C*Las

Multiple defense genes in HLB-tolerant citrus have been identified by multi-omics approaches (Table 2), although just the function of *Constitutive disease resistance* (*CDR*) and Non-expressor of *Pathogenesis Related genes 1* (*NPR1*) was confirmed in surrogate models or citrus.

*Constitutive disease resistance* genes belong to the plant aspartic proteinase (APs) gene family. *CDR1* was first identified and cloned in *Arabidopsis*. Its product has been implicated in disease resistance signaling (Xia et al., 2004). Overexpression of a rice (*Oryza sativa* L) *CDR 1* gene, led to constitutive activation of defense response and enhanced resistance in rice and *Arabidopsis* against bacterial and fungal pathogens (Prasad et al., 2009). Several studies have demonstrated that *CDR1* as potential candidate genes for HLB tolerance in *Poncirus* (Albrecht and Bowman, 2012; Du et al., 2015; Rawat et al., 2015). A study was undertaken to mine and characterize the *CDR* gene family in Citrus and *Poncirus* and to understand its association with HLB tolerance in *Poncirus*. It found that *PtCDR2* and *PtCDR8* were high abundance in *Poncirus* leaf transcriptomes. The expression of *PtCDR2* and *PtCDR8* genes responded to *C*Las infection differently in HLB-tolerant and susceptible genotypes (Rawat et al., 2017). The role of *PtCDR2* and *PtCDR8* in disease resistance was confirmed in *Arabidopsis* mutants that showed that transformation of *PtCDR2* and *PtCDR8* into *Arabidopsis cdr1* mutant induced *PR1* expression and recovered the hypersensitive response to *Pseudomonas syringae* pv. *tomato* strain DC3000 (Ying et al., 2020). Therefore, *PtCDR2* and *PtCDR8* play a key role in plant defense responses and serve as strong candidate genes for engineering citrus for HLB disease tolerance.

Non-expressor of *Pathogenesis Related genes 1* gene is a key regulator in the signal transduction pathway that leads to SAR response. The *NPR1* gene may act as a regulator of the transcription factor/s that controls *PR* gene expression (Kinkema et al., 2000) and mediates the salicylic acid (SA) induced expression of *PR* genes and SAR (Clarke et al., 1998). Plants over expressing *NPR1* display enhanced resistance to several pathogens (Cao et al., 1998). For instance, transcriptome profiling of HLB-tolerant “Jackson” (grapefruit hybrid) and HLB-susceptible “Marsh” grapefruit found that four *NPR1*-like genes were significantly upregulated in HLB tolerant citrus trees (Wang et al., 2016). Furthermore, transgenic sweet orange cultivars “Hamlin” and “Valencia” expressing an *A. thaliana npr1* gene under the control of a constitutive CaMV 35S promoter or a phloem specific *Arabidopsis* SUC2 (*AtSUC2*) promoter resulted in trees with normal phenotypes that exhibited enhanced resistance to HLB. Additionally, the transgenic trees exhibited reduced diseased severity and a few lines remained disease-free even after 36 months of planting in a high-disease pressure field site (Dutt et al., 2015). *AtNPR1* can enhance expression of transcription of genes encoding pathogen-associated molecular patterns (PAMPs), transcription factors, leucine-rich repeat receptor kinases (LRR-RKs), and putative ankyrin repeat-containing proteins, in *AtNPR1* transgenic line compared to the control plant (Qiu et al., 2020). These results suggested that *NPR1* positively regulates the innate defense mechanisms in citrus, contributing to enhance tolerance to citrus HLB.

## Activation of Antimicrobial Metabolites

Plants have a number of unique defense mechanisms including physical barriers to pathogen invasion as well as a wide range of secondary metabolites and antimicrobial peptides (AMP). Secondary metabolites have long been suggested to interact with pathogen (Hartmann, 2008). Several studies have revealed a vast number of secondary metabolites with proven or putative functions in plant responses to pathogen microorganisms (Piasecka et al., 2015). In several HLB-tolerance citrus cultivars, the transcriptomic analysis reveals that most differentially expressed genes (DEGs) increase in secondary metabolites pathways from HLB-tolerant citrus including *Poncirus trifoliata* and its hybrids (Albrecht and Bowman, 2012), “Jackson” (grapefruit hybrid; Wang et al., 2016), Mexican lime (*Citrus aurantifolia*; Arce-Leal et al., 2020), and Kaffir lime (*Citrus hystrix*; Zou et al., 2019). The secondary metabolites are higher in HLB-affected tolerant citrus cultivars, indicating a strong relationship between HLB-tolerance and secondary metabolites accumulation (Rao et al., 2018). In addition, amino acids including phenylalanine, tyrosine, and tryptophan were accumulated on HLB-tolerant citrus relative *P. trifoliata* (Killiny and Hijaz, 2016), which are involved in synthesis of many secondary metabolites. Furthermore, several studies demonstrated that HLB-tolerant citrus including US-942 (*P. trifoliata×Citrus reticulata*), Curry leaf [*Murraya koenigii* (L.) Spreng], and LB8-9 Sugar Belle contained high level of secondary metabolites such as volatile organic compounds (VOC), phenolics, and flavonoids (Killiny et al., 2017; Hijaz et al., 2020; Deng et al., 2021; Huang et al., 2021b).

Volatile organic compounds play a key role in protecting plants under insect and pathogen attack. VOCs, including aldehydes, monoterpenes, sesquiterpenes, thymol, b-elemene, and (E)-b-caryophyllene, have antimicrobial activities against pathogens, and accumulate in HLB-tolerant LB8-9 Sugar Belle (Killiny, 2017; Deng et al., 2021). Phenolics are a group of secondary metabolites, which are produced *via* the shikimic acid pathway through the phenylpropanoid pathway (Lin et al., 2016). It has been demonstrated that the accumulation of phenolic compounds at the infection site could result in pathogen restriction and prevention of their spread to other plant’s tissues (Nicholson and Hammerschmidt, 1992). Flavonoids are widely distributed in plants and they are synthesized in the cytosol through the phenylpropanoid pathway by a set of enzymes (Treutter, 2006; Petrussa et al., 2013). Flavonoids could exhibit their resistance to pathogens by inhibition and crosslinking of the microbial enzymes, chelation of metals necessary for enzyme activity, and formation of physical barrier (Treutter, 2006). The HLB-tolerant Curry leaf [*M. koenigii* (L.) Spreng] and LB8-9 Sugar Belle contain high level of phenolics and flavonoids, which correlate with their enhanced tolerance to *C*Las (Killiny et al., 2017; Hijaz et al., 2020). Therefore, increased levels of VOCs, phenolics, and flavonoids in citrus may contribute to HLB tolerance.

Antimicrobial peptides stand out as one of the most prominent components of the plant immune system. These small and usually basic peptides are deployed as a generalist defense strategy that grants direct and durable resistance against plant pathogens. A recent study identified a novel class of heat stable antimicrobial peptides (SAMPs), from HLB-tolerant citrus Australian finger lime (*Microcitrus australiasica*). SAMPs not only effectively reduced *C*Las titer and disease symptoms in HLB-positive trees but also prevented and inhibited infections by induction of defense response genes such as *PR1* and *PR2*, an enzyme of SA biosynthesis, phenylpropanoid pathways, and phenylalanine ammonia-lyase 1(PAL; Huang et al., 2021a). Thus, HLB-tolerant citrus can also be a source of defense peptides against *C*Las.

## Host Defense Triggered by Agents

Citrus defense mechanisms not only can be activated by pathogens, but also induced by agents such as chemical compounds, heat and nutrients. Systemic acquired resistance (SAR) can be useful to control of several plant diseases (Ryals et al., 1996; Sticher et al., 1997; Durrant and Dong, 2004). SAR involves in a specific defense signaling pathway that required SA and is associated with accumulation of pathogenesis-related proteins (PR). Several chemical compounds can activate SAR in plant. Four SAR activators including SA, oxalic acid, acibenzolar-S-methyl, and potassium phosphate, provided significant control of HLB by suppressing *C*Las titer and disease progress when applied by trunk injection (Hu et al., 2018). Furthermore, both SA and acibenzolar-S-methyl significantly induced expression of *PR-1* and *PR-2* genes, and oxalic acid and potassium phosphate resulted in significant induction of *PR-2* and *PR-15* gene expression, respectively (Hu et al., 2018). In addition, foliar spray application of several plant defense inducers [i.e., β-aminobutyric acid (BABA), 2,1,3-benzothiadiazole (BTH), 2,6-dichloroisonicotinic acid (INA), and ascorbic acid (AA)] were reported to suppress progress of HLB in the field. BTH and INA, which are functional analogs of SA, can induce plant defenses in citrus. The effect control of BABA on citrus HLB may be in SA-depend pathway. Furthermore, AA may alleviate HLB symptoms by interfering with biosynthesis of plant hormones (including salicylic acid and jasmonic acid; Li et al., 2016). Other plant hormones, such as brassinosteroids, can induce plant defenses against a wide range of pathogens including *C*Las. Foliar spray of brassinosteroid (24-epibrassinolide) in greenhouse and field experiments of HLB-affected citrus showed *C*Las titer was reduction after treatment under both conditions (Canales et al., 2016). Moreover, several chemical compounds have antimicrobial activities against *C*Las, and can also induce plant defense against the pathogen. Sulphonamide antibiotics such as sulfadimethoxine sodium (SDX) and sulfathiazole sodium (STZ) have been proved to be effective against *C*Las (Zhang et al., 2014; Yang et al., 2016a). Transcriptomic analysis of citrus plants revealed that SDX can induce genes related to the metabolism of jasmonates, brassinosteroids, ROS, and secondary metabolites, which are beneficial for resistance against HLB (Yang et al., 2020b; Table 3).

**Table 3:** Chemicals and heat activate citrus defense response.

Agent types	Name	Mechanisms against *C*Las	References
Chemicals	Salicylic acid	Induction of expression of *PR-1* and *PR-2* genes	Hu et al., 2018
	Acibenzolar-S-methyl	Induction of expression of *PR-1* and *PR-2* genes	Hu et al., 2018
	Oxalic acid	Induction of *PR-2* gene expression	Hu et al., 2018
	Potassium phosphate	Induction of *PR-15* gene expression	Hu et al., 2018
	β-Aminobutyric acid	Involving in SA-depend pathway	Li et al., 2016
	2,1,3-Benzothiadiazole	Functional analogs of SA	Li et al., 2016
	2,6-Dichloroisonicotinic acid	Functional analogs of SA	Li et al., 2016
	Ascorbic acid	Interfering with biosynthesis of plant hormones and the signaling process	Li et al., 2016
	24-Epibrassinolide	Induction of some plant defense genes such as glutathione peroxidase, Jasmonate acid	Canales et al., 2016
	Sulfadimethoxine sodium	Induction of genes related to the metabolism of jasmonates, brassinosteroids, reactive oxygen species (ROS), and secondary metabolites	Yang et al., 2020b
Heat	Solar thermotherapy	Many genes involved in plant-bacterium interactions being upregulated post treatment, which may be contributed to host defense against *C*Las	Doud et al., 2017
	Heat treatment (40°C)	A strong upregulation of chaperones involved in reversing the effects of *C*Las infection in citrus plants	Nwugo et al., 2016

*Candidatus* Liberibacter asiaticus is a heat-tolerant bacterium and can thrive under high temperature conditions extending to 35°C (Lopes et al., 2009). Many studies demonstrated that heat treatment (temperature ranged from 40 to 50°C) can eliminate or suppress *C*Las titer in HLB-affected citrus (Hoffman et al., 2013; Fan et al., 2016; Yang et al., 2016a,b). Moreover, the heat treatment also can enhance vigor of HLB-affected citrus and promote new flush (Hoffman et al., 2013; Yang et al., 2016b; Armstrong et al., 2021). Transcriptome analysis has shown that the gene expression profiles of HLB-affected trees post solar-heat treatment more closely modeled healthy trees than their gene profiles prior to treatment, with many genes involved in plant-bacterium interactions being upregulated post treatment, which may contribute to host defense against *C*Las (Doud et al., 2017). In addition, proteomics analysis indicated that a strong upregulation of chaperones including small (23.6, 18.5, and 17.9 kDa) heat shock proteins, a HSP70-like protein and a ribulose-1,5-bisphosphate carboxylase oxygenase (RuBisCO)-binding 60 kDa chaperonin, in response to heat treatment (40°C), which has been involved in reversing the effects of *C*Las infection in citrus plants (Nwugo et al., 2016; Table 3).

For several years, it has been reported that the application of enhanced nutritional products can extend the vigor of HLB-affected citrus and trigger citrus defense against *C*Las (Spann and Schumann, 2009; Shen et al., 2013; da Silva et al., 2020; Shahzad et al., 2020; Dong et al., 2021). Although nutrient treatments have no effect on reducing *C*Las titer and cannot enhance yield of HLB-affected citrus in the field (Gottwald et al., 2012; da Silva et al., 2020; Phuyal et al., 2020), the application of macro-and micronutrients have been adopted worldwide as they induce host defenses and help maintain production of HLB-affected trees. For instance, a field study in Florida showed application of phosphorus (P) oxyanion solutions to HLB-affected citrus mitigated disease symptom severity during a 3-year field trial (Zhao et al., 2013). It is known that phosphite has a direct action on plant defense mechanisms by the activation of the PAL activity and the biosynthesis of phytoalexins (Saindrenan and Guest, 1994), which may be involved in citrus defense induced by P. HLB-affected citrus display interveinal chlorotic leaves due to iron (Fe) deficiency caused by *C*Las (Masaoka et al., 2011) and foliar application of Fe^2+^ have shown to alleviate symptoms of HLB-affected citrus trees (Inoue et al., 2020). In other pathosystems, such as in rice-*Magnoporthe* interactions, rice plants growing at high Fe levels have enhanced resistance against the fungus. Although this has not been evaluated, application of Fe may induce host defense against *C*Las (Peris-Peris et al., 2017). Recent research indicates that elevated levels of manganese (Mn) promote better tree response to the effects of HLB increasing citrus tree lifespan (Morgan et al., 2016; Zambon et al., 2019). Sufficient Mn in the rhizosphere is critical for scavenging ROS (Alscher et al., 2002), which is known to be produced extensively in *C*Las-damaged cells (Ma et al., 2022). Although many nutrients can mitigate symptoms of HLB-affected trees, the mechanisms of how these nutrients trigger citrus defenses are still unclear and warrant investigation.

## Role of Citrus Microbiome in Combatting *C*Las

The plant microbiome is an important contributor to plant health and defense against pathogens. Plant-associated microbiota can suppress pathogens through direct competition, producing antimicrobial compounds or stimulating plant immunity to resist or tolerate pathogen infection (Saikkonen et al., 2004; Kaul et al., 2016; Brader et al., 2017). To date, a plethora of studies have focused on deciphering the role of the citrus microbiome with the goal of identifying members of the microbial community associated with HLB and *C*Las suppression. However, comparison of microbiomes from healthy and HLB-affected citrus have shown that *C*Las affects the microbial community structure and reduce the putative beneficial microbe associations within citrus leaves and roots (Trivedi et al., 2011; Zhang et al., 2017; Ginnan et al., 2020; Yan et al., 2021).

For example, *C*Las infection in mandarin leaves (*C. reticulata* cv. Shatangju) causes reduction of several beneficial bacteria genera including *Variovorax*, *Novosphingobium*, *Methylobacillus*, *Methylotenera*, and *Lysobacters*, which are known to be involved in promoting plant growth and antibiotic production (Yan et al., 2021). Study of Blaustein (2017)identified citrus-health-associated endophytes of leaves (such as *Methylpbacterium*, *Burkholderia*, and *Sphingomonas*) and roots (*Bradyrhizobiaceae*) based on increased relative abundances in healthy vs. HLB-diseased citrus trees. These potential beneficial microbes are known to be involved in competing with pathogens for nutrients, antagonize pathogens through antibiosis, assist the host with nutrient acquisition, and induce host defense responses (Compant et al., 2005; Madhaiyan et al., 2006; Enya et al., 2007; Lugtenberg and Kamilova, 2009; Verma et al., 2010; Innerebner et al., 2011; Ardanov et al., 2012). However, their reduction in HLB-affected citrus provides insights into the role of the microbial community into HLB progression.

Interestingly, inoculations of *Burkholderia* stains isolated from the rhizosphere of healthy citrus roots can induce the expression of genes involved in activation of citrus defenses and SA mediated induced systemic resistance (Zhang et al., 2017). Other studies have shown that *Bacillus* sp. can also induce host defense responses against *C*Las through enhancing expression of several transcription factors involved in disease resistance (Tang et al., 2018; Munir et al., 2020). Moreover, when the biocontrol agent *Xylella fastidiosa* strain EB92-1 was applied to HLB-affected citrus plants, the results indicated that it could reduce the incidence of HLB symptoms in mature trees through 18 months after inoculation and the incidence of severe symptoms up to 3 years (Hopkins and Wall, 2021). Although the mechanism of HLB suppression by *X. fastidiosa* EB92-1 and the other bacteria remain to be studied in depth, these studies show that beneficial bacteria can be used to suppress *C*Las and improve plant health by induction of plant defenses that confer broad-spectrum resistance against pathogens.

Manipulation of the citrus microbiome to enrich the populations of beneficial microbes in HLB-affected trees could aid in disease suppression. Actually, nutrients play a role in activating plant immunity system by altering the microbial community structure and the metabolism (Sugimoto et al., 2010; Shi et al., 2012; Huber and Jones, 2013). A recent study has reported that application of calcium, magnesium, and boron to the soil can alter microbial structure and communities in phyllosphere and rhizosphere of HLB-affected citrus and promoted beneficial microorganism (*Burkholderiaceae*, *Xanthomonas*, and *Stenotrophomonas*) enrichment, which may have contributed to the reduced HLB incidence, and *C*Las titers (Zhou et al., 2021).

Chemotherapy is another method that can shape the citrus microbiome. Antimicrobial activity of the effective antibiotics against *C*Las have been associated with shifts in endophytic microbial structure and communities in HLB-affected citrus after treatment (Zhang et al., 2014; Yang et al., 2015, 2020a; Li et al., 2019). Foliar application of penicillin and oxytetracycline to HLB-affected citrus, caused an increase in the relative abundance of beneficial bacterial species, including *Streptomyces avermitilis* and *Bradyrhizobium*, compared to those treated with water control (Yang et al., 2020a). Moreover, the relative abundance of the bacterial species associated with *C*Las survival, such as *Propionibacterium acnes* and *Synechocystis* sp. PCC 6803, was lower in penicillin and oxytetracycline treated plants compared to the control (Yang et al., 2020a). Other studies have shown that the endophytic microbiome was altered in HLB-affected scion treated with ampicillin, and 10 abundant operational taxonomic units (OTUs) from antibiotic producing *Stenotrophomonas* spp. were only detected in the ampicillin-treatment (Zhang et al., 2013). Study of Ascunce et al. (2019) also showed that Bacilli, involved in the elicitation of plant defenses against pests and pathogens, were relatively more abundant in petioles and roots from penicillin treated HLB-affected citrus. Moreover, it was also found that the endophytic microbiome was changed in HLB-affected citrus plants under heat and sulfonamide (sulfathiazole sodium–STZ, and sulfadimethoxine sodium—SDX) treatments (Yang et al., 2016a). Following antibiotic treatment with SDX and STZ, there was enhanced abundance of OTUs belonging to the families *Streptomycetaceae*, *Desulfobacteraceae*, *Chitinophagaceae*, and *Xanthomonadaceae*, which are beneficial for control of plant pathogens and promoting plant growth (Hell, 1997; Kim and Jung, 2007; Mhedbi-Hajri et al., 2011; Mendes et al., 2013). Therefore, the enrichment of beneficial bacteria in these antibiotic treatments, may be contributed to their antimicrobial activity against *C*Las.

It is clear that the citrus microbiome plays a key role in citrus health. Whether some bacteria have an effect in survival of *C*Las is still unclear. The enrichment of beneficial bacteria in healthy citrus or in response to effective chemical compounds, may be involved in combating *C*Las. However, more studies are needed to validate the role of beneficial bacteria in citrus, and identify antagonistic bacteria against *C*Las (Trivedi et al., 2011; Riera et al., 2017; Zhang et al., 2017). Therefore, isolation and identification of the enriched beneficial bacteria can provide more insight into the role of citrus microbiome in HLB mitigation.

## New Approaches for Studying Mechanisms Against Uncultured Bacterial Pathogens

Despite the advances in uncovering virulence mechanisms of *C*Las, identification of genes conferring disease tolerance, discovering potential antagonistic bacteria, and identifying many small molecules that inhibit *C*Las, we are still far from deploying sustainable solutions to the HLB epidemic.

Establishing *C*Las in culture can provide an extended vision in mechanism of agents against *C*Las. Although several reports of transient *C*Las cultures have been published, most of these attempts have only been able to maintain *C*Las in coculture (Davis et al., 2008; Parker et al., 2014; Fujiwara et al., 2018; Ha et al., 2019; Merfa et al., 2019). These studies partially fulfilled Koch’s postulates and could potentially be used to unravel the complex relationships of *C*Las with other citrus endophytes; however, no follow-up research using these approaches to obtain a pure *C*Las culture has been published. Currently, the methods employed to study the mode of action of small molecules with antimicrobial activity have been elucidated *in vitro* using as culturable surrogate models such as *L. crescens* and *S. meliloti* (Pagliai et al., 2014; Barnett et al., 2019). However, all sequenced *C*Las strains have reduced genome size of about 1.2 Mb (Duan et al., 2009; Thapa et al., 2020), compared with the slightly larger 1.5 Mb of *L. crescens* BT-1 (Leonard et al., 2012), and the about 6.7 Mb genome of the phylogenetically related *S. meliloti* (Sugawara et al., 2013) which could cause differences in biosynthetic pathways, metabolic enzymes, and secretion systems. Therefore, novel approaches are needed to uncover how chemicals, nutrition, beneficial microorganisms or hosts, directly or indirectly affect *C*Las. One such approach is the recent development of a plant hairy root system that mimics the host environment and supports the growth of *C*Las (Irigoyen et al., 2020). This system was developed as a tool for high throughput screening of antimicrobials against *C*Las and *Candidatus* Liberibacter solanacearum (CLso), which is faster and more reliable compared to conventional compound screening approaches (Irigoyen et al., 2020). Thus, this system could also be used as a model to study the mode of action of antimicrobials against *C*Las inside the citrus host.

Uncovering the complex interactions of *C*Las and the host, is key to discover pathways that can be exploited for disease suppression. However, genome-wide transcriptome profiling of a phloem-restricted pathogen in planta is very difficult, since the bacterial mRNA constitutes a minor fraction of the total mRNA. Thus, most research has focused on gene expression of the citrus host, and smaller number of studies describe global gene expression profiles of the pathogen. To examine the expression profiles of *C*Las in the host, most studies rely on quantitative reverse transcription-polymerase chain reaction approaches which only address targeted genes. *In vivo* transcriptomic analyses are required to understand the active pathways in *C*Las. A recent study identified the regions in the citrus fruit pith with higher bacterial titers which was used to conduct RNA-seq analysis after rRNA removal (Fang et al., 2021). This study compared the gene expression profiles of the fruit pit vs. leaf midribs and found different gene expression profiles related to virulence genes; however, the resolution of the transcriptome profile was lower in midribs compared to fruit pit mainly due to the lower bacterial titers (Fang et al., 2021).

Because the main limitation of conducting transcriptomic profiles of *C*Las is bacterial titers, different enrichment approaches are being developed. A *C*Las enrichment system using dodder showed about 419-fold *C*Las titer increase in dodder system as compared to the corresponding citrus hosts, and the dual RNA-seq data indicated that similar *C*Las gene expression profiles in dodder and citrus samples, yet dodder samples generated a higher solution than those obtained in citrus host (Li et al., 2021b). Although the *C*Las-enrichment dodder system could be used as surrogate model for studying interaction of *C*Las and host, dodder defense system against *C*Las is very different from citrus. To overcome the limitation of surrogate systems, a bacterial cell enrichment procedure has been developed for transcriptome profiling of *C*Las in citrus in which bacteria is isolated from citrus samples prior to RNA extraction, reaching detectable expression to 84% of the *C*Las genome coverage (De Francesco et al., 2022). This *C*Las-enrichment method will be useful for mechanisms of *C*Las within the citrus host and for elucidating potential targets for *C*Las suppression.

## Conclusion

The mechanisms to study phloem-limited and uncultured plant bacterial pathogens are a complicated process. The development of novel approaches to understand the virulence mechanisms of the pathogen, the mode of action of antimicrobial therapies, the interactions with host and other endophytic microbes will aid in the search of effective and sustainable methods to combat *C*Las and ultimately HLB.

**Figure 2:**
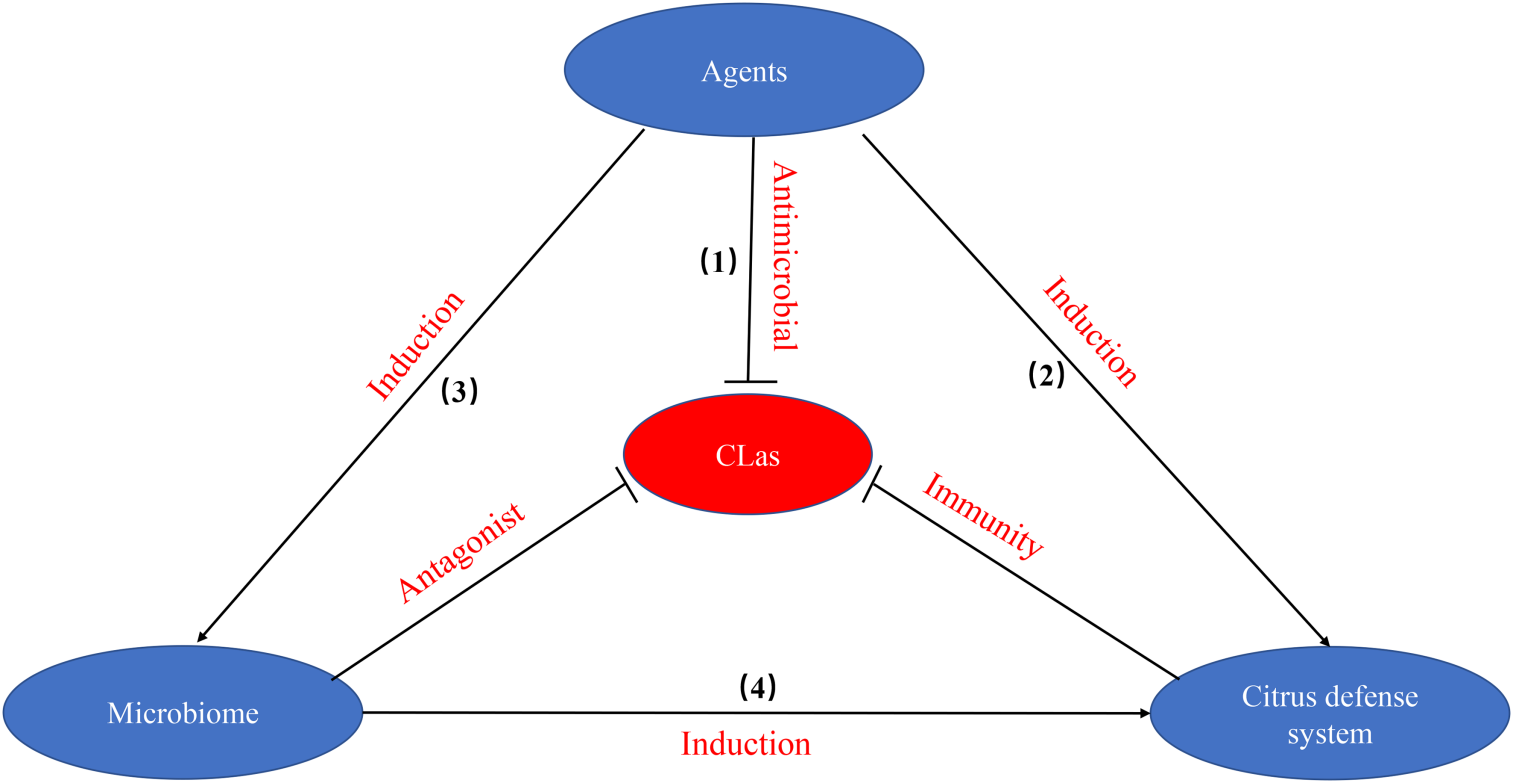
The pathways involved in effective agents including chemicals, nutrition, and plant defense activators against CLas.

Until we unravel the mechanistic black box in the interactions between citrus phloem and *C*Las, the combination of effective agents including chemicals, nutrition, and plant defense activators will continue to be the only path to combat HLB (Figure 2). To effectively combat HLB, multiple strategies need to be applied against *C*Las: (1) the use of antimicrobial agents that directly disturb the biological processes of *C*Las, thus affecting bacterial survival; (2) the use of chemical agents that suppress *C*Las by inducing citrus host defense systems; (3) modifying the environment by agents to promote the enrichment of beneficial bacteria to antagonize *C*Las; and (4) the enrichment of beneficial bacteria that trigger citrus defense system against *C*Las. These pathways may work separately or together to promote tree health, mitigate HLB and recover tree productivity. Therefore, the relationship of virulence targets, citrus defenses and microbiome plays a key role in elucidating mechanisms against *C*Las.

## Author Contributions

CY and VA contributed to writing and editing this manuscript. All authors read and approved the final version of the manuscript.

## Funding

This study was supported by funds from the USDA-NIFA-SCRI (2019-70016-29096, 2018-70016-28198, and 2016-70016-24833) to VA.

## Conflict of Interest

The authors declare that the research was conducted in the absence of any commercial or financial relationships that could be construed as a potential conflict of interest.

## Publisher’s Note

All claims expressed in this article are solely those of the authors and do not necessarily represent those of their affiliated organizations, or those of the publisher, the editors and the reviewers. Any product that may be evaluated in this article, or claim that may be made by its manufacturer, is not guaranteed or endorsed by the publisher.
